# Digitale Dermatologie

**DOI:** 10.1111/ddg.70365

**Published:** 2026-07-07

**Authors:** Lea Henkel, Avend Bamarni, Stephan Alexander Braun, Valentina Busik, Holger Andreas Haenssle, Stephan Rietz, Paul Schmidle, Sandra Schuh, Anna‐Theresa Seitz, Sebastian Sitaru, Stephan Traidl, Max Tischler, Felix von Krogh, Julia Welzel, Julia Winkler, Anastasia Sophie Vollmer, Katharina Susanne Kommoss, Alexander Zink

**Affiliations:** ^1^ Klinik und Poliklinik für Dermatologie und Allergologie am Biederstein TUM School of Medicine and Health Technische Universität München München Deutschland; ^2^ Klinik für Dermatologie und Venerologie Universitätsklinikum Freiburg, Medizinische Fakultät, Albert‐Ludwigs‐Universität Freiburg Freiburg Deutschland; ^3^ Christine Kühne‐Center for Allergy Research and Education (CK‐CARE) Davos Schweiz; ^4^ Abteilung für Dermatologie, Medizinische Fakultät, Universität Münster Münster Deutschland; ^5^ Abteilung für Dermatologie, Medizinische Fakultät Heinrich‐Heine‐Universität Düsseldorf Deutschland; ^6^ Praxis für Dermatologie und Allergologie, Dr. med. Thomas Führer Gießen Deutschland; ^7^ Abteilung für Dermatologie, Universität Heidelberg Heidelberg Deutschland; ^8^ Hautklinik und Poliklinik, Universitätsmedizin Mainz Deutschland; ^9^ Klinik für Dermatologie und Allergologie Universitätsklinikum Augsburg Augsburg Deutschland; ^10^ Klinik und Poliklinik für Dermatologie, Venerologie und Allergologie, Universitätsklinikum Leipzig AöR Hannover Deutschland; ^11^ Klinik für Dermatologie, Allergologie und Venerologie Medizinische Hochschule Hannover Hannover Deutschland; ^12^ Facharzt für Dermatologie, Haut+Laserpraxis Dr. Tischler+Team Dortmund Deutschland

**Keywords:** Digitalisierung, Dermatologie, Künstliche Intelligenz, Big Data, Teledermatologie, Patientenzentrierte Versorgung, Mobile Anwendungen, Interoperabilität, Datensicherheit, Digitalization, Dermatology, Artificial Intelligence, Big Data, Teledermatology, Patient‐centered care, Mobile applications, Interoperability, Data security

## Abstract

Die digitale Transformation verändert die Dermatologie grundlegend und eröffnet neue Möglichkeiten in Diagnostik, Therapie und Versorgungsorganisation. Große Datenmengen in Kombination mit Künstlicher Intelligenz (KI) erlauben eine präzisere Klassifikation und Prognose, insbesondere durch die Analyse klinischer und dermatoskopischer Bilddaten. Zunehmend werden auch synthetisch generierte Daten für das Training neuer Algorithmen genutzt, deren klinische Validität noch geprüft werden muss. Teledermatologie hat sich als fester Bestandteil der Versorgung etabliert. Videokonsultationen und asynchrone Bildübertragungen verbessern die Erreichbarkeit dermatologischer Expertise und könnten insbesondere in strukturschwachen Regionen eine Versorgungslücke schließen. Mobile Anwendungen und digitale Plattformen fördern zudem Adhärenz, Selbstmonitoring und die aktive Beteiligung der Patientinnen und Patienten am Behandlungsprozess. Trotz dieser Chancen bestehen gleichzeitig auch große Herausforderungen. Datenschutz, Interoperabilität und regulatorische Rahmenbedingungen müssen adressiert werden, um eine nachhaltige Implementierung sicherzustellen. Entscheidend ist darüber hinaus die interdisziplinäre Zusammenarbeit zwischen Medizin, Technik und Gesundheitsökonomie. Dabei kommt Ärztinnen und Ärzte als Fachexperten eine zentrale Rolle zu, sowohl bei der Bewertung der Datenqualität als auch bei der klinischen Interpretation digitaler Systeme. Ebenso wichtig ist eine kontinuierliche Schulung des Fachpersonals. Insgesamt bietet die Digitalisierung ein großes Potenzial zur Verbesserung der dermatologischen Versorgung. Voraussetzung hierfür sind strukturierte Prozesse, Qualitätssicherung und die konsequente Einbindung von Patientinnen und Patienten.

## EINLEITUNG

Die Digitalisierung ist längst kein Zukunftsthema mehr, sondern prägt bereits heute die dermatologische Versorgung. Kaum ein anderes Fach lebt so stark von visueller Diagnostik, definierten Prozessen und interdisziplinären Schnittstellen ‐ Eigenschaften, die die Dermatologie besonders für digitale Innovationen prädestinieren.^1^

Von künstlicher Intelligenz (KI)‐gestützter Bildanalyse über telemedizinische Anwendungen, die elektronische Patientenakte (ePA) und Big‐Data‐Ansätze bis hin zu interaktiven Tools wie Wearables oder Avataren entstehen neue Versorgungsmodelle. Diese fördern die aktive Einbindung von Patientinnen und Patienten in Prävention und Therapie und eröffnen durch kontinuierliches Monitoring zugleich Perspektiven für eine stärker individualisierte und ortsunabhängige Versorgung.

Gleichzeitig bringt der digitale Wandel auch Herausforderungen mit sich. Fragen der Validierung, Ethik, des Datenschutzes und der praktischen Integration in den Klinik‐ und Praxisalltag müssen beantwortet werden, bevor neue Technologien ihr volles Potenzial entfalten können. Für Ärztinnen und Ärzte bedeutet dies, neben dem klassischen Fachwissen auch digitale Kompetenzen aufzubauen und in den klinischen Alltag zu integrieren.^2^

Zur fachlichen Einordnung und Begleitung dieser Entwicklungen haben sich innerhalb der Dermatologie verschiedene Initiativen und Netzwerke etabliert, darunter auch der Arbeitskreis Digitale Dermatologie innerhalb der Deutschen Dermatologischen Gesellschaft (DDG), der den interdisziplinären Austausch und den Wissenstransfer zu digitalen Anwendungen fördert.

Dieser CME‐Artikel bietet einen Überblick über die zentralen Entwicklungen, die derzeit die digitale Transformation in der Dermatologie in Deutschland prägen. Er spannt den Bogen von den Grundlagen der Datennutzung und künstlicher Intelligenz über konkrete klinische Anwendungen bis hin zu patientenzentrierten Tools und Fragen der Versorgungsorganisation. Diese spiegeln nicht nur aktuelle Forschung und Praxis wieder, sondern geben auch einen Ausblick auf zukünftige Entwicklungen.

## GRUNDLAGEN UND DATENBASIS

### Big Data

Der Begriff Big Data ist in den letzten Jahren im medizinischen Kontext zunehmend in den Fokus gerückt. Ursprünglich durch die IT‐Analystengruppe Gartner geprägt, beschreibt er große Datenmengen (Volumen / Volume), eine hohe Entstehungs‐ und Verarbeitungsgeschwindigkeit (Verarbeitungsgeschwindigkeit / Velocity) sowie eine große Datenvielfalt (Vielfalt / Variety), die mit klassischen Methoden nicht effizient verarbeitet werden können. Für den medizinischen Bereich reicht diese Definition jedoch nicht aus, da hier zusätzlich die Datenqualität (Wahrhaftigkeit / Veracity) sowie der potenzielle Nutzen für die Patientinnen‐ und Patientenversorgung und die Forschung (Wert / Value) eine entscheidende Rolle spielen.^3^

Big Data umfasst strukturierte und unstrukturierte Daten aus unterschiedlichen Quellen: elektronische Patientenakten, Bilddaten, molekulargenetische Profile, patientenbasierte Messwerte, Daten aus Apps und Wearables oder auch Informationen aus sozialen Netzwerken. Diese Daten können im Rahmen eines Datenlebenszyklus erhoben, verarbeitet, analysiert und in medizinische Entscheidungsprozesse überführt werden.

Big Data ist mehr als nur Datenmenge – es beschreibt ein komplexes Zusammenspiel aus Datengewinnung, Analyse und Anwendung mit dem Ziel u. a. einer verbesserten medizinischen Versorgung.

Es ist zudem wichtig, zwischen datenintensiven Anwendungen und dem Einsatz maschinellen Lernens zu unterscheiden. Nicht jede Big‐Data‐Anwendung nutzt KI, und nicht jedes KI‐Modell benötigt große Datenmengen.

Die Dermatologie eignet sich besonders gut für datenbasierte Anwendungen. Visuelle Diagnostik, chronische Verläufe und standardisierte Scores bieten eine gute Grundlage für strukturierte Datenerfassung. Register wie TREATgermany oder PsoBest ermöglichen die Erfassung realer Versorgungssituationen, die jenseits klinischer Studien wichtige Erkenntnisse liefern. Dazu gehören beispielsweise Daten zur Therapietreue, zur Patientenstratifizierung, zum langfristigen Nutzen von Biologika oder zur Entwicklung der Lebensqualität unter Systemtherapie.^4^, ^5^ Darüber hinaus können dermatologische Bilddaten automatisiert analysiert werden.

Allerdings zeigt sich in der praktischen Umsetzung, dass die Nutzung von Big Data mit erheblichen Herausforderungen verbunden ist. Eine zentrale Voraussetzung für belastbare Analysen ist die strukturierte und qualitätsgesicherte Dokumentation medizinischer Informationen. Schon kleinere Inkonsistenzen oder fehlende Standards können zu fehlerhaften Schlussfolgerungen führen. Das Grundprinzip „garbage in, garbage out“ (GIGO) beschreibt dieses plakativ: Nur aus hochwertigen Eingangsdaten können valide Ergebnisse generiert werden. ^6^

Big Data ist nur so gut wie die zugrunde liegenden Daten – Struktur, Standardisierung und Qualität sind essenziell für belastbare Ergebnisse.

Eine weitere Voraussetzung für die nachhaltige Nutzbarkeit medizinischer Daten sind die sogenannten FAIR‐Prinzipien. Diese fordern, dass Daten auffindbar (findable), zugänglich (accessible), interoperabel (interoperable) und wiederverwendbar (reusable) sein müssen. In der dermatologischen Praxis betrifft dies insbesondere die digitale Dokumentation, die semantische Standardisierung klinischer Parameter sowie die technische Anschlussfähigkeit an andere Systeme.^7^

Auch der Datenschutz stellt eine zentrale Herausforderung dar. Die Verarbeitung personenbezogener Gesundheitsdaten muss im Einklang mit der Datenschutzgrundverordnung erfolgen. Dabei ist insbesondere die Wahrung der informationellen Selbstbestimmung von Bedeutung. Patientinnen und Patienten müssen nachvollziehen können, zu welchen Zwecken ihre Daten verwendet werden. Transparente Einwilligungsprozesse, klare Zweckbindung und technische Sicherheitsstandards sind unverzichtbar.^8^

Die Nutzung algorithmischer Verfahren birgt zusätzlich das Risiko systematischer Verzerrungen. Solche Verzerrungen können durch unausgewogene Stichproben (sampling bias), fehlerhafte Zieldefinitionen (label bias) oder unzuverlässige Eingangsdaten (measurement bias) entstehen. In der dermatologischen Bildanalyse ist insbesondere die unzureichende Repräsentation dunkler Hauttypen ein bekanntes Problem. Hinzu kommt, dass viele Modelle wie neuronale Netze oder Support Vector Machines eine geringe Nachvollziehbarkeit für Anwenderinnen und Anwender aufweisen, was die klinische Bewertung erschweren und die Akzeptanz mindern kann.

Unter diesen Herausforderungen eröffnet Big Data in der Dermatologie vielfältige Anwendungsfelder: von der automatisierten Diagnostik über die individualisierte Therapiebegleitung bis hin zu populationsbezogenen Präventionsansätzen. Damit dieses Potenzial Eingang in die klinische Praxis findet, sind klar definierte methodische, rechtliche und ethische Rahmenbedingungen erforderlich, ebenso wie interoperable IT‐Strukturen, standardisierte Dokumentationsprozesse und eine enge Zusammenarbeit zwischen medizinischen Fachdisziplinen, Datenwissenschaften und gesundheitsbezogenen Entscheidungsträgern.^3^, ^7^

Die fundierte Auseinandersetzung mit digitalen Technologien und datenbasierten Instrumenten wird zunehmend Teil ärztlicher Verantwortung. Dies erfordert eine kontinuierliche Weiterentwicklung der Kompetenz im Umgang mit Gesundheitsdaten, um die Versorgungsqualität im digitalen Wandel aktiv mitzugestalten.

Die bloße Existenz dieser umfangreichen Datenpools generiert jedoch noch keinen unmittelbaren klinischen Nutzen. Um aus der Rohdatenmenge handlungsrelevantes Wissen zu extrahieren, bedarf es leistungsfähiger Analysewerkzeuge, wie beispielsweise Klassifikationsalgorithmen.

### Klassifikation von Groß und Klein: Klinische und Labor‐KI in der Dermatologie

Während Big Data die Grundlage für datengetriebene Anwendungen liefert, wird der tatsächliche Mehrwert erst durch geeignete Auswertungsverfahren wie Klassifikationsalgorithmen sichtbar. In der Dermatologie lassen sich damit sowohl Laborprozesse als auch klinische Anwendungen unterstützen.

Klassifikationsalgorithmen der KI haben insbesondere in der Unterscheidung maligner und benigner Hauttumoren ein hohes diagnostisches Potenzial gezeigt.^9^ Darüber hinaus eröffnen KI‐basierte Ansätze auch Anwendungsmöglichkeiten in weiteren Bereichen der Dermatologie. So kann beispielsweise das Wissen von Experten in der Auswertung nativer mykologischer Proben, etwa Tesafilmabrissen, in KI‐Algorithmen überführt werden.^10^

Am anderen Ende des Datenspektrums stehen klinische Bilder und Ganzkörperaufnahmen.^11^ KI kann hier unter anderem eine automatische Zuordnung zu Körperregionen ermöglichen,^12^ um beispielsweise unstrukturierte Bilddatenbanken Big‐Data‐Analysen zugänglich zu machen. Dabei kann ein einmal erstellter Datensatz auch für zukünftige, meistens performantere Algorithmen eingesetzt werden.^10^

Die Dermatologie bietet ideale Voraussetzungen für KI‐gestützte Klassifikation: Bild‐, dermatoskopische und histopathologische Daten liegen in großer Zahl vor und sind vergleichsweise leicht zu standardisieren.

Aus methodischer Sicht ist zu beachten, dass Klassifikationsalgorithmen in der Regel nur für spezifische Datentypen trainiert sind und daher nur bei passendem Input valide Ergebnisse liefern. Die Klassifizierung von (Bild‐)Daten stellt dabei einen bereits gut erschlossenen Einsatzbereich von KI in der Medizin und insbesondere in der Dermatologie dar, wie zum Beispiel für die Entscheidung benigne/maligne bei dermatoskopischen Aufnahmen.^9^ Aktuelle Entwicklungen in den Computerwissenschaften fokussieren sich neben großen Sprachmodellen (large language model, LLM) unter anderem auf Algorithmen zur Generierung von neuen (synthetischen) Bilddaten,^13^ die beispielswiese auch für die Dermatologie nutzbar gemacht werden könnten. Erste Ansätze, synthetische Daten zu generieren, um zum Beispiel die Datensätze für Klassifikationsalgorithmen zu ergänzen, wurden bereits mit Erfolg in der Informatik‐Literatur publiziert.^14^

Während sich Klassifikationsalgorithmen primär auf die strukturierte Verarbeitung einzelner Datentypen ausrichten, charakterisiert die nächste Entwicklungsstufe den Übergang zu Systemen mit höherem Autonomiegrad, den sogenannten KI‐Agenten.

KI‐Algorithmen benötigen für valide Ergebnisse spezifische Datentypen und eine hohe Standardisierung der Inputs.

### AI agents und agentic AI

Die bisherigen KI‐Systeme in der Dermatologie sind meist auf klar abgegrenzte Aufgaben spezialisiert und erreichen insbesondere in der Befundung bestimmter histologischer Diagnosen eine hohe diagnostische Genauigkeit.^15^ Spätestens seit Ende 2022 ist mit der allgemeinen Verfügbarkeit eines populären LLM eine stärker generalistisch ausgelegte KI im Bewusstsein von Ärztinnen, Ärzten sowie Patientinnen und Patienten angekommen. Mittlerweile können diese Programme in unterschiedlichem Ausmaß auch Bilder, Audio‐ und Videomaterial verarbeiten, „verstehen“ und sogar erzeugen (generatives KI‐Modell).

Bislang erfordern generative KI‐Systeme (z. B. ChatGPT, Claude, Gemini) eine aktive Steuerung durch den Anwender mittels spezifischer Eingabebefehle, sogenannter „Prompts“.

Eine automatisierte Übernahme solcher Aufgaben könnte daher einen nächsten Entwicklungsschritt darstellen. Dazu bedarf es eines Systems, das eine Vielzahl heterogener Daten versteht und diese korrekt verarbeiten kann.

In einem solchen Szenario könnten Laborberichte gemeinsam mit weiteren Laborwerten strukturiert abgelegt werden, während das System auffällige Befunde unmittelbar an den menschlichen Nutzer meldet. Parallel ließen sich ärztliche Befunde analysieren, potenzielle Inkonsistenzen mit aktuellen Daten für eine spätere Prüfung gekennzeichnen und anschließend systematisch archivieren.

KI Agenten sind generative KI‐Systeme, die eigenständig Entscheidungen treffen und Handlungen ausführen können, um Ziele zu erfüllen. Diese können je nach Design des Systems alleine handeln (single‐agent) oder im Team Aufgaben aufteilen (multi‐agent‐system).

Für all diese Schritte bedarf es eines zugrundeliegenden generativen KI‐Modells, das Bild und Sprache verarbeiten kann. Zusätzlich ist jedoch ein Grad an Autonomie erforderlich und hier kommen AI‐agents (deutsch: KI‐Agenten) ins Spiel. Ein AI‐agent ist ein auf einem KI‐Modell basierendes Computerprogramm, das eigenständig mit seiner Umgebung interagieren kann, um ein vorgegebenes oder selbstdefiniertes Ziel zu erfüllen.^16^

Dazu sind folgende grundlegende Komponenten nötig:

**Table:** 

Ein geeignetes KI‐Modell	AI‐model
Erinnerung, Speicher: die Fähigkeit, sich Abläufe und den Kontext der Handlungen merken zu können.	memory
Planendes Vorgehen: Aufgaben in Teilaufgaben oder Handlungsschritte aufteilen, diese priorisieren und die passenden Teilschritte zur Lösung koordinieren.	planning, reasoning
Werkzeuge: die Möglichkeit, auf Datenquellen (zum Beispiel E‐Mails, Arztbriefe, aktuelle Leitlinien) zugreifen und Programme zur Datenverarbeitung (zum Beispiel MS Excel, Praxisverwaltungssoftware) nutzen zu können.	tools
Selbstkontrolle: Aufgabenfortschritt und Zwischenergebnisse müsse kontrolliert und wenn nötig geändert werden, wenn neue Erkenntnisse dies erfordern.	state‐management

**ABBILDUNG 1:**
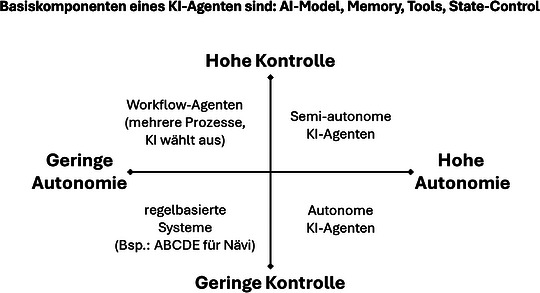
Arten von KI‐Agenten in Abhängigkeit von Autonomie und menschlicher Kontrolle. Die Grafik verdeutlicht das Spannungsfeld zwischen der Eigenständigkeit eines Systems (Autonomie) und dem Grad der direkten Steuerung durch den Nutzer (Kontrolle). Während regelbasierte Systeme eine geringe Autonomie aufweisen, agieren autonome KI‐Agenten weitgehend selbstständig bei gleichzeitig reduzierter direkter Kontrolle.

Im ärztlichen Umfeld ist die Fähigkeit zur Kommunikation und Interaktion mit menschlichen Nutzern (user‐interface, human‐in‐the‐loop) wünschenswert, um die Aufgaben des Agenten zu überwachen und eine sinnvolle Zusammenarbeit zu ermöglichen. Der entscheidende Baustein, der ein Softwareprogramm eigenständig handeln lässt, ist das Ausmaß an eigenständigem Handeln (autonomy), das erlaubt wird (Abbildung 1). Eine Abwägung zwischen Autonomie und Kontrolle ist nötig.

Aktuell gibt es noch keine evidenzbasierten Untersuchungen zum Einsatz von KI‐Agenten in der Dermatologie. Agentische KI, also KI‐Systeme, die eigenständig mit ihrer Umgebung interagieren, um vorgegebene oder selbstdefinierte Ziele zu erreichen, könnte in Zukunft unter anderem in folgenden Szenarien eingesetzt werden:

- **Triage**: Automatische Bewertung von Hautveränderungen, aktive Rückfragen an Patientinnen und Patienten und Anfordern von Vorbefunden. Synthese der Informationen, Erkennen von dringenden Befunden.
- **Monitoring**: Therapiekontrolle durch Überprüfen von Patientenfotos, Erinnerung an Folgetermine und nötige Laborkontrollen
- **Semi‐autonomes Therapiesystem**: Aktive Kontrolle des Therapieerfolgs von Biologika bei Psoriasis anhand validierter Fragebögen, Anfordern von Verlaufsfotos von Patientinnen und Patienten, Führen eines Patientenportals und Kontrolle von gemeldeten Nebenwirkungen. Bei Abweichung vom Therapieziel empfehlen geeigneter Maßnahmen anhand geltender Leitlinien.

Bis zur flächendeckenden Einführung agentischer KI gilt es, qualitativ hochwertige Forschung zu liefern, damit die Agenten den Anwendern nutzen, Arbeit erleichtern und Gesetzesvorgaben eingehalten werden. Die agentischen KI‐Systeme mit medizinischer Zweckbestimmung sind nach der EU MDR in der Regel als Software als Medizinprodukt einzustufen und unterliegen einer risikoadäquaten Konformitätsbewertung.^17^ Der EU AI Act klassifiziert viele KI‐Systeme im Gesundheitswesen als Hochrisiko‐KI‐Systeme und fordert unter anderem ein Risikomanagement, eine Daten‐ und Modellgovernance, Transparenz, menschliche Aufsicht sowie ein Post‐Market‐Monitoring.^18^

Der Einsatz digitaler Technik erfordert auch bei KI‐Agenten zunächst die genaue Definition des Problems, bevor über die Sinnhaftigkeit, Effizienz und Wirtschaftlichkeit eines Systems entschieden werden kann. Ebenso entscheidend ist es, die Arbeitsschritte und Prozesse sowie die Kommunikationsschnittstellen im Arbeitsalltag zu kennen, um einen KI‐Agenten sinnvoll zur Arbeitserleichterung in den Ablauf einbinden zu können. Bisherige Erfahrungen zeigen, dass analoge Arbeitsabläufe häufig lediglich digital abgebildet („elektrifiziert“) werden, anstatt die Prozesse für die digitale Umgebung neu zu konzipieren. Für eine erfolgreiche Integration von KI‐Agenten ist ein grundlegendes Prozess‐Redesign (Process Re‐Engineering) ein zentraler Erfolgsfaktor.^19^, ^20^

## KLINISCHE ANWENDUNG VON KI

Nach der Darstellung der grundlegenden Konzepte von Big Data und Klassifikationsalgorithmen richtet sich der Blick nun auf deren konkrete Einsatzmöglichkeiten in der dermatologischen Praxis. Besonders bildgebende Verfahren wie Dermatoskopie, LC‐OCT (Line‐Field Confocal Optical Coherence Tomography) oder die digitale Pathologie zeigen, wie KI‐Systeme diagnostische Prozesse unterstützen, die Genauigkeit erhöhen und Arbeitsabläufe effizienter gestalten können. Gleichzeitig werden Limitationen deutlich, die eine verantwortungsvolle Einbettung dieser Technologien in den klinischen Alltag erfordern.

### Leistung und Limitierung von KI‐basierten Systemen in der Dermatoskopie

Die Dermatologie bietet sich aufgrund ihres visuell‐morphologischen Schwerpunktes für „sehende“ künstliche Intelligenz (KI)‐Systeme in besonderem Maße an.^21^, ^22^ Als besonders geeignet für diesen Bereich der „computer vision“ haben sich künstliche neuronale Netzwerke (KNN) erwiesen.^23^, ^24^ KNN sind in der Lage nach einem umfangreichen Training anhand von klinischen oder dermatoskopischen Bildern eine Wahrscheinlichkeit für eine bestimmte Diagnose anzugeben.^25^ Das Training entspricht dabei einem klassischen Lernen am Beispiel, oder anders ausgedrückt einem „Repräsentationslernen“.^23^ Für die korrekte Erkennung eines malignen Melanoms bedeutet dies, dass im Training möglichst viele Unterformen des Melanoms vertreten sein sollten (d.h. auch noduläre, akrale, oder amelanotische Melanome).^26^ Im Training muss ein KNN also anhand tausender Bilder lernen, welche morphologischen Merkmale ein Melanom ausmachen, und wie sich diese von Merkmalen anderer Hauttumore unterscheiden.^27^

Vor der Einführung von KNN wurden auch bereits Computersysteme in der Hautkrebsdiagnostik eingesetzt.^28^ Diese basierten auf einer einfachen, von Experten programmierten Merkmalerkennung, und zeigten im Vergleich zu den heutigen KNN‐basierten Systemen eine deutlich geringere diagnostische Leistungsfähigkeit.^29^ Für die Diagnose des Melanoms, wurde damals die Erkennung von Asymmetrie, Anzahl an Farben, Beschaffenheit der Begrenzung und der Durchmesser der Läsion programmiert. Verständlicherweise konnten bei diesem „regelbasierten“ Ansatz niemals alle Subtypen und Sonderformen erfasst werden, was letztendlich zu einem Verlust an diagnostischer Genauigkeit führte.

Nachdem erste Machbarkeitsstudien den Nutzen von KNN‐basierten Systemen bei der Unterscheidung von Melanomen und Nävi auf dem Niveau ausgebildeter Dermatologinnen und Dermatologen demonstrierten,^9^ konzentrierte sich die Forschung in den folgenden Jahren vor allem auf die Ausweitung des Trainings auf weitere benigne und maligne Hautläsionen. Heutige konkurrenzfähige KNN‐basierte Systeme sind in der Regel für die Erkennung der folgenden diagnostischen Klassen trainiert: Melanom (MEL), Basalzellkarzinom (BCC), Plattenepithelkarzinom (SCC), aktinische Keratose und Morbus Bowen (ACIEK), melanozytärer Nävus (NV), Dermatofibrom (DF), benigne vaskuläre Tumore (VASC), und seborrhoische Keratose und Lentigo senilis (BKL). Auch nach Ausweitung des Trainings auf diese zusätzlichen Klassen zeigte sich in Studien die diagnostische Trefferquote weiterhin auf dem Niveau von Hautfachärztinnen und Hautfachärzten.^30^ Eine erste prospektive „real‐world“ Studie zur Zusammenarbeit von „Mensch und Maschine“ bei der Erkennung der Melanom‐Diagnose lieferte zudem überzeugende Daten mit verbesserter Sensitivität und Spezifität durch die KI‐Unterstützung.^31^

Eine vergleichende Studie zu einem KNN‐basierten System und einem „regelbasierten“ System ergab einen eindrucksvollen Unterschied mit einer rund 20 % höheren diagnostischen Leistung des KNN‐basierten Systems.^29^

**ABBILDUNG 2:**
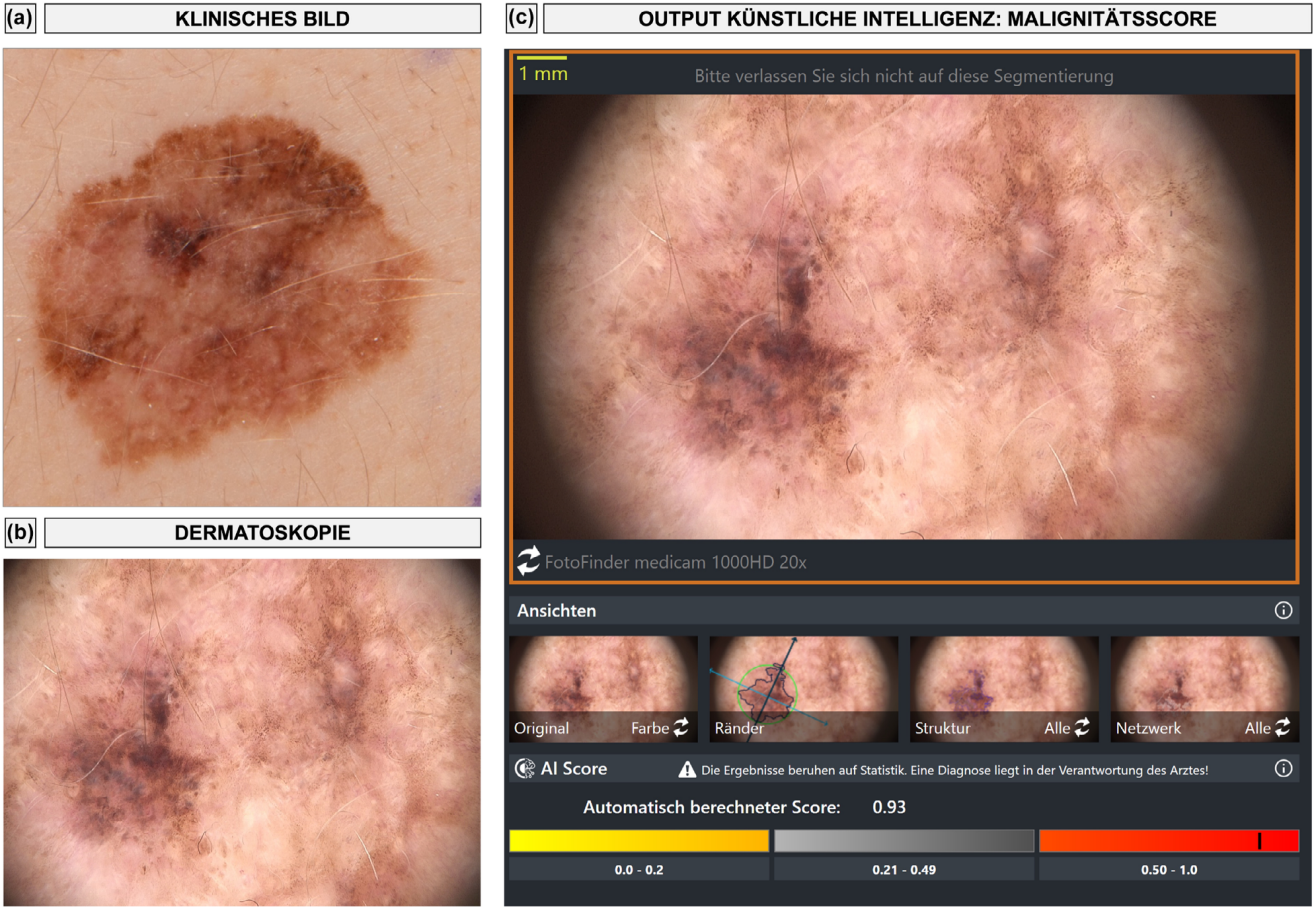
KI‐gestützte Malignitätsbewertung eines superfiziell spreitenden Melanoms. (a) Klinische Nahaufnahme eines superfiziell spreitenden Melanoms mit unregelmäßigen Rändern und verschiedenfarbiger Pigmentierung (Tumordicke 0,6 mm) am Rücken eines 53‐jährigen Patienten. (b) Dermatoskopie (20‐fache Vergrößerung, polarisiertes Licht) mit Zeichen der immunologischen Regression und (c) des Melanophagenreichtums. Der berechnete Score liegt bei einer Malignitätswahrscheinlichkeit von 0,93 bei einem Grenzwert für Malignität von ≥ 0,5 (FotoFinder Systems GmbH, Bad Birnbach)

Abbildung 2 zeigt exemplarisch das Ergebnis einer KI‐Untersuchung eines malignen Melanoms. Bei diesem Beispiel wurde eine Malignitätswahrscheinlichkeit von 0,93 (d. h. 93 %) ausgegeben (Werte ≥ 0,5 deuten auf eine wahrscheinlich maligne Läsion hin).

Generell scheinen dermatoskopische Bilder, gegenüber klinischen Übersichtsbildern oder Nahaufnahmen von Hautläsionen, häufiger zu korrekten Diagnosen zu führen, da detailreichere Aufnahmen („feature‐rich“) mehr diagnostisch verwertbare Merkmale enthalten.^21^, ^32^

Des Weiteren müssen „Expertensysteme“ für die Anwendung durch Ärztinnen und Ärzte mit entsprechender medizinischer Zulassung im Rahmen des Medizinproduktegesetztes von „Laiensystemen“ (meist Smartphone APPs) für die Nutzung durch Patientinnen und Patienten unterschieden werden. Die obigen Ausführungen beziehen sich dabei auf Expertensysteme, da die Datenlage für „Laiensystem“ insgesamt als sehr unbefriedigend bezeichnet werden muss.^33^ Entweder deuten vorliegenden Studiendaten auf eine unzureichende diagnostische Leistung hin oder fehlen gänzlich.

Beim klinischen Einsatz von KNN‐basierten Diagnosesystemen ist die Kenntnis potenzieller Limitationen und Fallstricke essenziell. Neuronale Netzwerke mit medizinischer Zulassung sind Assistenzsysteme und liefern für eine abschließende Diagnose, welche in der Verantwortung der Ärztin oder des Arztes liegt, nur einen einzelnen Teilaspekt. Darüber hinaus machen KNN‐basierte Systeme in der Regel keine Angaben dazu, mit welcher Sicherheit oder Unsicherheit eine (korrekte oder falsche) Diagnose gestellt wurde.^34^ Bildartefakte wie farbliche Markierungen auf der Haut können die Leistungsfähigkeit von KNN‐Systemen signifikant beeinträchtigen und sollten strikt vermieden werden.^35^ Eine weitere Limitation ergibt sich durch einen Mangel an Trainingsbildern für seltene Diagnosen, welche das Spektrum der oben genannten häufigeren diagnostischen Klassen verlassen (zum Beispiel kutane Lymphome, seltene Adnextumore, kutane Sarkome, oder seltene Unterformen anderer Tumore).

Zusammenfassend lässt sich feststellen, dass KI‐basierte Diagnosesystem für die Hautkrebsdiagnose in der Dermatologie angekommen sind. Weitere prospektive Studien sind notwendig, um den Nutzen solcher Systeme und weitere Einsatzgebiete noch besser zu erforschen. Während die Dermatoskopie den etabliertesten Einsatzbereich für KI darstellt, eröffnen neue nichtinvasive Bildgebungsverfahren wie die LC‐OCT zusätzliche diagnostische Möglichkeiten.

Für eine zuverlässige KI‐Diagnostik müssen im Training möglichst alle klinischen Unterformen einer Entität (z. B. noduläre oder amelanotische Melanome) vertreten sein.

### Integration von KI in die nichtinvasive Bildgebung

Die präzise Diagnose von Hauterkrankungen wie aktinischer Keratose (AK), Basalzellkarzinom (BCC) und Psoriasis, ihre Quantifizierung und Verlaufsbeobachtung ist in der klinischen Praxis oft eine Herausforderung. Konventionelle Methoden wie klinische Untersuchung oder Dermatoskopie liefern wertvolle Hinweise, erreichen jedoch bei unklaren Läsionen schnell ihre Grenzen. Biopsien bleiben der diagnostische Goldstandard, sind jedoch invasiv und potenziell belastend für die Patientinnen und Patienten (v. a. an delikaten Lokalisationen). Aus diesen Gründen gewinnen nicht‐invasive diagnostische Verfahren zunehmend an Bedeutung.

Mit der Line‐field konfokalen optischen Kohärenztomographie (LC‐OCT) steht ein bildgebendes Verfahren zur Verfügung, das mikroskopieähnliche, dreidimensionale Echtzeitaufnahmen der Haut liefert – ohne Gewebeentnahme.^36^, ^37^ In Kombination mit KI entstehen dabei objektive, reproduzierbare Aufnahmen, die sowohl zur Erstdiagnose als auch zur Verlaufskontrolle herangezogen werden können. KI‐Algorithmen erkennen charakteristische Merkmale wie epidermale Veränderungen und atypische Zellkerne.^38^

In der AK‐Diagnostik erlaubt die LC‐OCT die Visualisierung typischer histologischer Merkmale wie Hyperkeratose, Parakeratose und Atypien ohne Gewebeentnahme.^39^, ^40^ KI‐gestützte Analysen, etwa des PRO‐Scores zur Einschätzung der basalen epidermalen Proliferation, erlauben eine feinere Risikoeinschätzung und erleichtern die Überwachung des Therapieerfolgs unter nichtinvasiven Therapieoptionen.^41^, ^42^

**ABBILDUNG 3:**
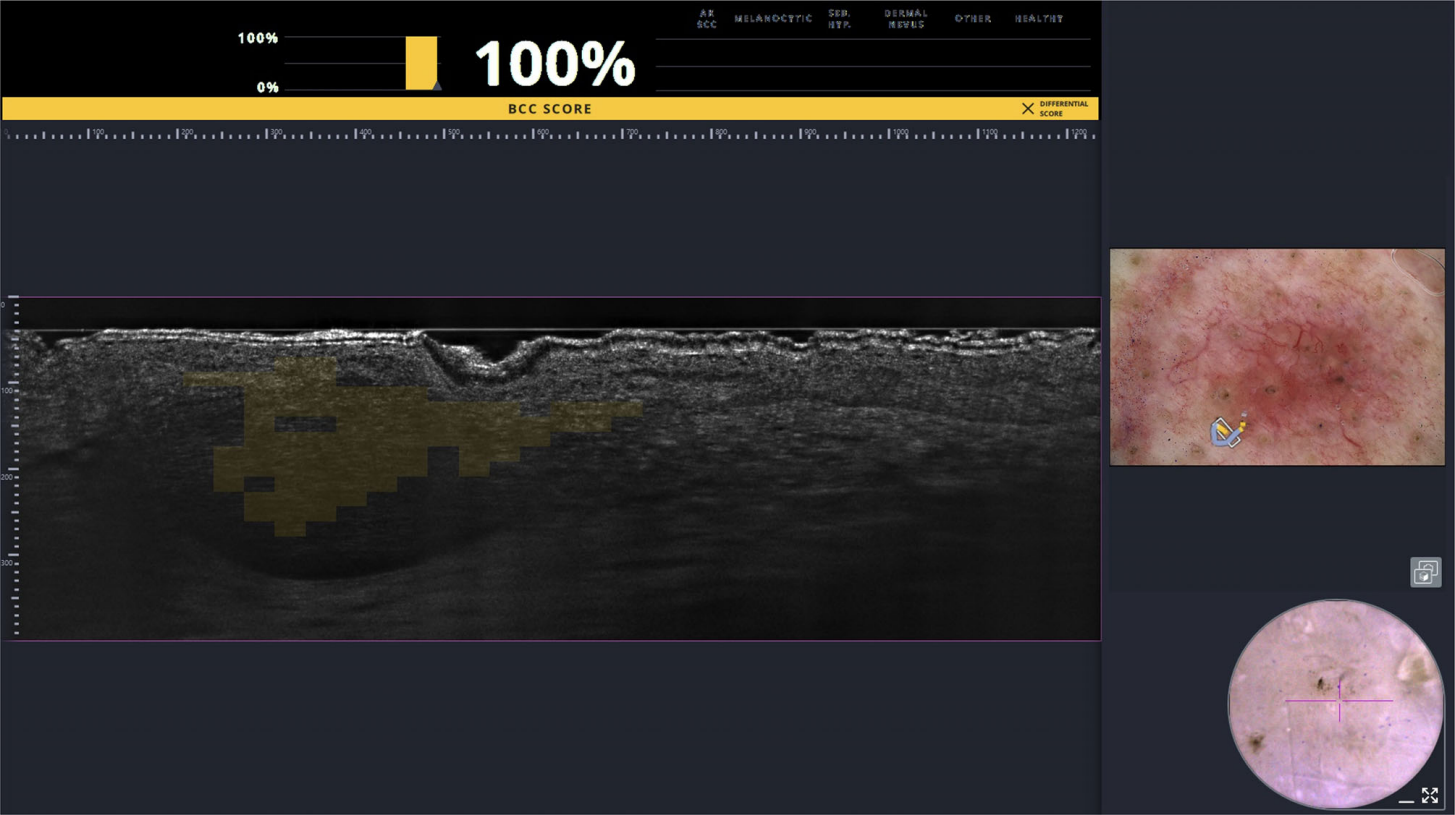
Line‐field konfokale optische Kohärenztomographie (LC‐OCT) eines nodulären Basalzellkarzinoms (BCC) am Rücken mit KI‐gestützter Analyse. Die Abbildungen zeigen sowohl die LC‐OCT‐Querschnitte als auch die durch die KI erzeugte Überlagerung, welche potenziell tumorverdächtige Strukturen hervorhebt. Gelb markierte Segmente kennzeichnen Bereiche, die vom Algorithmus mit sehr hoher Wahrscheinlichkeit (nahezu 100 %) als charakteristisch für ein BCC eingestuft werden. Blaue Markierungen hingegen stehen für Regionen mit sehr geringer Trefferwahrscheinlichkeit (nahe 0 %). Die Farbskala orientiert sich am prognostizierten KI‐Score und reicht von blau (0 %) bis gelb (100 %). Neben der numerischen Wahrscheinlichkeitsangabe am Bildrand erstellt das Modell eine Heatmap, die über die LC‐OCT‐Daten gelegt wird und farbige Felder sichtbar macht, die auf mögliche typische BCC‐Muster hinweisen. Im rechts oben gezeigten Übersichtsbild ist die gesamte Läsion dargestellt. Durch die Kolokalisationssoftware wird exakt aufgezeigt, an welchen Stellen innerhalb der Läsion noch tumorverdächtiges Gewebe (gelb) vorhanden ist und wo kein Hinweis auf Tumorareale besteht (blau). Auf diese Weise lassen sich die LC‐OCT‐Schnittbilder präzise mit der lokalen Tumorverteilung verknüpfen. Links unten befindet sich das entsprechende Dermatoskopiebild, das eine makroskopische Orientierung bietet und die räumliche Zuordnung der LC‐OCT‐Scans innerhalb der Läsion ermöglicht.

Darüber hinaus besteht ein großer Nutzen bei der BCC‐Diagnostik: Ein mit histologisch referenzierten Bilddaten trainierter Deep‐Learning‐Algorithmus kann in Echtzeit zwischen BCC und Differenzialdiag‐nosen unterscheiden. Farblich codierte Heatmaps markieren verdächtige Areale direkt im Bild (Abbildung 3), was die diagnostische Sicherheit erhöht – vor allem bei weniger erfahrenen Ärztinnen und Ärzten.^43^

Außerdem kann eine KI‐gestützte Randmarkierung von BCC bereits vor der Operation helfen, die Tumoren genau zu kennzeichnen.^44^, ^45^

Multizentrische Untersuchungen belegen eine signifikante Verbesserung von Sensitivität und Spezifität in der Detektion von BCC mit der LC‐OCT durch KI‐Unterstützung im Vergleich zur allein klinischen und dermatoskopischen Diagnostik. ^43^

Mit der LC‐OCT steht ein nichtinvasives bildgebendes Verfahren zur Verfügung, welches mittels einer integrierten erklärbaren KI in Echtzeit in den Bildern Heatmaps mit Wahrscheinlichkeit einer Diagnosestellung darstellt.

Auch in der Psoriasis‐Diagnostik eröffnet die KI‐gestützte LC‐OCT neue Möglichkeiten: Veränderungen der Epidermis‐ und Stratum‐corneum‐Dicke lassen sich millimetergenau messen und über den Therapieverlauf dokumentieren.^46^, ^47^ Diese objektiven Parameter korrelieren eng mit dem klinischen Ansprechen. Während sich epidermale Parameter oft rasch normalisieren, bleiben vaskuläre Veränderungen teils länger bestehen – ein potenzieller Marker für persistierende Krankheitsaktivität.^46^

Die Integration in den klinischen Alltag erfordert jedoch klare Standards für Bildaufnahme, Datenverarbeitung und Ergebnisinterpretation. Ebenso sind rechtliche und ethische Fragen zu klären, insbesondere im Hinblick auf Datenschutz und ärztliche Verantwortung. KI sollte stets als Assistenzsystem verstanden werden – die finale Diagnosehoheit bleibt in ärztlicher Hand.

Zukunftsweisend ist das Potenzial, invasive Eingriffe zu reduzieren, Diagnosen und Operationen zu beschleunigen und die dermatologische Versorgung personalisierter zu gestalten. Neben der Praxisrelevanz eröffnet diese Technologie auch neue Perspektiven für die Forschung, etwa zur detaillierten Analyse der Tumor‐ und Entzündungsbiologie.^48^

Während LC‐OCT den diagnostischen Alltag ergänzt, verändern digitale Workflows und KI zunehmend auch die histopathologische Diagnostik im Labor.

### Digitalisierung in der Dermatopathologie

Durch den rasanten technischen Fortschritt hält auch in der Dermatopathologie die Digitalisierung zunehmend Einzug, und das gleich auf mehreren Ebenen.

Zum einen lassen sich organisatorische Prozesse durch einen digitalen Workflow, der von der Probenerfassung über das Labormanagement bis hin zur digitalen Befundübermittlung und ‐archivierung sämtliche Laborschritte umfasst,^49^, ^50^ deutlich effizienter gestalten.^51^

Die digitalisierten Glasobjektträger werden in speziellen Image‐Management‐Systemen dargestellt. Diese ermöglichen nicht nur die gleichzeitige Ansicht verschiedener Färbungen und den Vergleich mit klinischen Bildern oder anderen Informationen, sondern auch das schnelle Annotieren interessanter Strukturen im Gewebeschnitt. So können diese später für Besprechungen oder Diskussionen schneller wiedergefunden werden. Zusätzlich können WSI einfacher digital ausgetauscht und mit Experten weltweit diskutiert werden.^52^

Durch sogenannte Whole‐Slide‐Image(WSI)‐Scanner lassen sich gesamte histologische Schnitte einfach, schnell und in hoher Qualität digitalisieren und völlig neue Formen der Schnittbeurteilung schaffen.

Ferner steigert die durch die Digitalisierung entstehende räumliche Unabhängigkeit die Attraktivität des Berufsbildes, da nun auch flexible Arbeitsmodelle (z. B. Homeoffice) realisierbar sind.^53^ Angesichts der sinkenden Zahl praktizierender Pathologen,^54^ und des stetig wachsenden Probenaufkommens ist es heute wichtiger, denn je Wege zu finden, um engagierten Nachwuchs für die Pathologie zu begeistern.

Auch die dermatopathologische Ausbildung kann heute problemlos online und standortunabhängig erfolgen. Der Münsteraner Histologiekurs im neuen digitalen Format,^55^ und die Onlineplattform der Arbeitsgemeinschaft Dermatologische Histologie (ADH) mit ihren seit Jahren erfolgreichen digitalen Schnittseminaren sind dafür repräsentative Beispiele.^56^

Der digitale Gewebeschnitt bildet die Grundlage für den Einsatz von künstlicher Intelligenz (KI), die auch in der Dermatopathologie in den letzten Jahren zunehmend erforscht und entwickelt wurde. Ein Schwerpunkt liegt auf KI‐Assistenzsystemen, die Pathologen bei zeitaufwendigen Routineaufgaben unterstützen sollen. Studien belegen bereits das Potenzial, etwa bei der Detektion von Pilzelementen in Nagelmaterial,^57^, ^58^ oder der automatisierten Erkennung, Subtypisierung und Tumordickenmessung von Basalzellkarzinomen.^59^ Erste dieser Systeme, beispielsweise für Ki‐67, Her2, ER/PR oder PD‐L1, sind in der EU bereits CE‐zertifiziert und damit in der Routinediagnostik nutzbar.

Eine neue Entwicklung sind Vision‐Language‐Modelle, die Bild‐ und Textinformationen kombinieren und als erweiterte Assistenzsysteme eingesetzt werden. In ersten Studien können sie bereits Fragen zu histopathologischen Schnitten beantworten,^60^ oder automatisiert Befundtexte erstellen, etwa beim Basalzellkarzinom.^61^

Darüber hinaus wird erforscht, wie KI subvisuelle Gewebemerkmale identifizieren kann, die als digitale Biomarker dienen könnten.^62^, ^63^, ^64^ Diese Verfahren sind jedoch noch nicht Teil der Routine.

KI‐Assistenzsysteme können Pathologen bei zeitaufwendigen Routineaufgaben unterstützen, wie der Detektion von Pilzelementen oder der automatisierten Tumordickenmessung von Basalzellkarzinomen.

Dennoch existieren Faktoren, die einer raschen, flächendeckenden Digitalisierung in der Dermatopathologie entgegenstehen. Neben hohen Anschaffungskosten für Hard‐ und Software sowie dem großen Bedarf an Speicherkapazität und Energie, ist hier vor allem der hohe Zeitaufwand zu nennen, den die Umstrukturierung über Jahre gewachsener Laborstrukturen erfordert.^65^, ^66^ Auf Seiten von KI‐Algorithmen stellt sich zudem die Frage nach der diagnostischen Qualität, für die in der Entwicklung eine hervorragende Datenqualität essenziell ist und die damit unweigerlich verbundenen rechtlichen und ethischen Fragen der Nutzung.

Viele dieser Hürden scheinen überwindbar, sodass auch die Zukunft der Dermatopathologie digital sein wird.

## DIGITALE TOOLS UND PATIENTENZENTRIERUNG

Während sich die bisherigen Beispiele vor allem auf diagnostische und laborbasierte Anwendungen konzentrieren, gewinnen im nächsten Schritt vor allem patientennahe digitale Werkzeuge an Bedeutung. Technologien wie Wearables (körpernahe digitale Messsysteme), Avatare (digitale, interaktive Schnittstellen zur Patientenkommunikation) oder digitale Kommunikationsplattformen verlagern den Fokus von der reinen Diagnostik hin zur aktiven Einbindung der Patientinnen und Patienten in Prävention, Therapie und Versorgungsprozesse.

### Wearables

Ein zentrales Beispiel für patientennahe digitale Technologien sind Wearables, also körpernah getragene elektronische Systeme, die kontinuierlich gesundheitsrelevante Daten erfassen und Patientinnen und Patienten aktiv in Prävention und Krankheitsmanagement einbinden.

Miniaturisierte, hautnahe Systeme erfassen kontinuierlich Parameter wie Temperatur, Feuchte, Druck, pH oder transepidermalen Wasserverlust und können, je nach Sensorik, sogar Biomarker im Schweiß (Elektrolyte, Metabolite, Proteine, Hormone) detektieren.^67^ Für die Dermatologie eröffnet dies neue Optionen für Monitoring und individualisierte Versorgung; longitudinal erhobene Daten erlauben es, Trigger, Krankheitsdynamik und Therapieeffekte alltagsnah abzubilden.^68^, ^69^ Bei entzündlichen Dermatosen wie der atopischen Dermatitis (AD) steht die Vorhersage von Exazerbationen im Fokus. Ein in Entwicklung befindlicher, hautnaher Sensor (Armband‐Formfaktor) kombiniert klassische Hautparameter mit elektrodermaler Aktivität (EDA) als Surrogat des sympathikotonen Stressniveaus – ein Trigger, der retrospektiv oft schwer zu greifen ist. Ergänzend wurden membranbasierte Schweiß‐Sensoren beschrieben, die Cortisol nicht‐invasiv messen; solche Stress‐Marker könnten mit klinischen Scores und Patient‐Reported Outcomes (PROs) verknüpft werden, um personalisierte Warnschwellen („digital biomarkers“) zu definieren. Ein aktuelles Beispiel für den Mehrwert kontinuierlicher, alltagsnaher Biosignale liefert eine vor kurzem publizierte Studie über multimodale Wearable‐Daten in der Schwangerschaft: Aus Ring‐Sensoren abgeleitete Temperatur‐, Herz‐ und Aktivitätsverläufe zeichneten hochauflösende Trajektorien von der Konzeptions‐ bis zur Postpartum‐Phase nach und identifizierten physiologische Abweichungen bei frühem Schwangerschaftsverlust. Diese Arbeit unterstreicht die Machbarkeit und klinische Relevanz hochfrequenter, realweltlicher Zeitreihen – ein Konzept, das sich auf dermatologische Langzeitverläufe übertragen lässt (z. B. Flare‐Vorhersage, Therapie‐Titration).^70^ Bestehende Limitationen umfassen höhere Einstiegshürden bei älteren Patientinnen und Patienten, eine generelle Technik‐ und Konnektivitätsabhängigkeit sowie den hohen Integrationsbedarf in bestehende Workflows. Entscheidend ist daher die Kopplung mit mHealth‐Apps als Schnittstelle zwischen Patientin und Patient, Device und Praxis oder Klinik – nicht als Ersatz, sondern als Ergänzung ärztlicher Versorgung. Für die Praxis empfiehlt sich ein stufenweises Vorgehen, beginnend mit der Definition eines klaren Use‐Cases (zum Beispiel AD‐Flare‐Monitoring) und relevanter Endpunkte (PROs, Exazerbationsrate). Es folgen die Klärung von Datenschutz und Einwilligung, die Schulung des Teams sowie abschließend die Evaluation im Routinebetrieb. So lässt sich der Mehrwert digitaler Dermatologie messbar und patientenzentriert realisieren.^71^

Wearables (z. B. Smartwatches) und „Smart‐Skin“‐Sensoren entwickeln sich von Lifestyle‐Gadgets zu klinischen Werkzeugen.

Während Wearables vor allem Daten für das Monitoring bereitstellen, stehen digitale Avatare für eine neue Form der Kommunikation und Aufklärung, die das Arzt‐Patienten‐Gespräch ergänzt.

### Avatargenerierte Patientenaufklärung

Patientenaufklärung ist eine zentrale ärztliche Pflicht und Voraussetzung für Vertrauen, Therapieadhärenz und informierte Entscheidungen. In der Dermatologie betrifft dies nicht nur größere Eingriffe wie die operative Versorgung, sondern auch kleinere Eingriffe, systemische Therapien und chronische Erkrankungen. Im klinischen Alltag sind die Rahmenbedingungen oft ungünstig: Ärztinnen und Ärzte arbeiten unter hohem Zeitdruck, während Patientinnen und Patienten nach belastenden Diagnosen emotional überfordert sein oder auf sprachliche Hürden stoßen können.^72^

Durch die Verwendung digitaler Avatare kann ein sicherer Raum geschaffen werden, in dem standardisierte, verständliche und mehrsprachige Aufklärungsinhalte in gleichbleibender Qualität bereitgestellt werden, wodurch das ärztliche Gespräch ergänzt, aber nicht ersetzt wird.

Unter einem medizinischen Avatar versteht man ein KI‐basiertes, digital erzeugtes Abbild einer ärztlichen Person oder eines ärztlich freigegebenen Kommunikationsmodells, bei dem Stimme, Mimik und Gestik realitätsnah dargestellt werden. Die Inhalte der Videos werden von der jeweiligen Praxis oder Klinik definiert und ärztlich geprüft. Patientinnen und Patienten können diese Informationen sowohl vor Ort als auch zu Hause abrufen, mehrfach ansehen und im eigenen Tempo verarbeiten, beispielsweise gemeinsam mit Angehörigen.

Technisch basieren solche Systeme auf großen Sprachmodellen (large language models, LLMs) und multimodalen KI‐Anwendungen, die Text in Sprache, Gesichtsausdrücke und Bewegungen übersetzen. Ein einmal erstellter Avatar kann so für eine Vielzahl standardisierter Aufklärungsvideos genutzt werden.^72^

Ein Beispiel ist die Aufklärung vor einer Operation bei Hautkrebs: Der Avatar erklärt Ablauf, Risiken und Nachsorge. Patientinnen und Patienten erhalten einen QR‐Code/Link und können die Informationen zu Hause erneut anschauen. Für Menschen mit begrenzter Deutschkenntnis ist entscheidend, dass die Inhalte in der eigenen Muttersprache verfügbar sind – eine Grundlage für echte Teilhabe.^73^

Viele Patientinnen und Patienten fühlen sich in der Informationsflut verloren: Fachbegriffe sind unverständlich, und nach einer Diagnose fehlt oft die emotionale Kapazität, komplexe Inhalte sofort zu verarbeiten. Avatare begegnen diesem Problem mit laiengerechter Sprache, Vermeidung von Fachwörtern sowie Untertiteln. Patientinnen und Patienten können die Videos beliebig oft ansehen, ohne die Hemmung, durch wiederholtes Nachfragen den Praxisablauf zu stören.

Aufklärungsgespräche beanspruchen oft mehr Zeit als die eigentliche Untersuchung – etwa bei Koloskopien oder Gastroskopien. Werden Anweisungen zur Vorbereitung nicht verstanden, muss die Untersuchung wiederholt werden. Avatare können dies verhindern, indem sie die Anleitungen klar vermitteln. Gleichzeitig reduzieren sie Rückfragen bei Hausärzten und MFAs und entlasten so das Versorgungssystem. Ärztinnen und Ärzte können sich dadurch stärker auf individuelle Rückfragen konzentrieren, anstatt die Standardaufklärung unter Zeitdruck durchzuführen.^72^

Ein aktuelles Beispiel ist das Projekt ArztAvatar.de. Ziel ist es, Ärztinnen und Ärzte in wenigen Minuten zu befähigen, mit eigenem Gesicht und Stimme einen personalisierten Avatar zu erstellen. Damit wird die ärztliche Kommunikation nicht ersetzt, sondern erweitert: Patientinnen und Patienten kommen besser vorbereitet ins Gespräch, Ärztinnen und Ärzte gewinnen wertvolle Zeit.^73^

Der Fokus liegt aktuell auf nicht‐interaktiven Formaten. An der Universitätsmedizin Mainz läuft hierzu eine klinische, fragebogenbasierte Studie mit Patientinnen und Patienten, die wegen eines Hautkrebses operiert werden: Die Kontrollgruppe erhält die Standardaufklärung, die Interventionsgruppe zunächst eine Avatar‐Aufklärung mit anschließender ärztlicher Besprechung. Ziel ist die Untersuchung von Verständnis, Akzeptanz und Zeitersparnis. Parallel wird ein Anamnese‐Avatar entwickelt, der strukturierte Befragung und Dokumentation übernehmen soll.

Darüber hinaus wird untersucht, wie Avatare in der Ausbildung genutzt werden können. Dafür werden virtuelle Patientinnen und Patienten mit unterschiedlichen Eigenschaften erstellt, etwa ängstlich, sprachlich eingeschränkt oder konfrontativ, um Medizinstudierende im Umgang mit kritischen Aufklärungssituationen zu schulen.

Avatare könnten nicht nur Patientinnen und Patienten unterstützen, sondern auch als Trainingsinstrument für künftige Ärztinnen und Ärzte dienen.

### Chancen

**Table:** 

**Standardisierung**	Inhalte sind medizinisch geprüft und in gleichbleibender Qualität verfügbar.
**Verständlichkeit**	Klare Sprache, Vermeidung von Fachjargon und Untertitel erhöhen das Verständnis erheblich.
**Vertrauen**	Durch die Verwendung von Gesicht und Stimme der behandelnden Ärztinnen und Ärzte bleibt die persönliche Beziehung gewahrt.
**Patient Empowerment**	Informationen sind pausierbar, wiederholbar, laienverständlich und in der Muttersprache verfügbar.
**Effizienz**	Ärztinnen und Ärzte sparen Zeit bei sich wiederholenden Standardaufklärungen und können sich stärker auf individuelle Fragen konzentrieren.
**Skalierbarkeit**	Ein Avatar kann hunderte Videos generieren, ohne Mehraufwand für die Praxis.

### Herausforderungen

**Table:** 

**Rechtlich**	Nach §630e BGB bleibt das mündliche Gespräch Pflicht. Avatare dürfen nur ergänzen.
**Datenschutz**	DSGVO‐Konformität, Standort der Server und die Vermeidung von unkontrollierter Modellweiterbildung mit Patientendaten sind kritisch.
**Akzeptanz**	Ältere oder digital unerfahrene Patientinnen und Patienten bevorzugen oft den persönlichen Kontakt und sind mit Video‐ oder Tablet‐Nutzung teils überfordert.

In den nächsten Jahren werden Avatare zu interaktiven, empathischen und adaptiven Systemen. Eingebettet in Telemedizin‐Plattformen, Klinik‐Apps oder Anamnese‐Stationen können sie ein wichtiger Bestandteil der Versorgung werden. Kombiniert mit KI‐gestützten Triage‐Systemen oder digitalen Patientinnen‐ und Patientenzwillingen entsteht so ein Ökosystem, das Ärztinnen entlastet und gleichzeitig die Patientinnen‐ und Patientenkommunikation verbessert.^72^, ^73^

Es eröffnet sich die Chance zu einer doppelten Transformation: mehr Zeit für die ärztliche Kernaufgabe und mehr Verständlichkeit für Patientinnen und Patienten.

Neben dieser strukturierten, ärztlich geprüften Kommunikation gewinnen jedoch auch unkontrollierte Informationskanäle wie Social Media zunehmend Einfluss auf das Gesundheitsverhalten.

Avatargenerierte Aufklärungsvideos ermöglichen Patienten das Verarbeiten komplexer Informationen im eigenen Tempo und in der jeweiligen Muttersprache – eine Voraussetzung für informierte Entscheidungen.

### Social Media und Wissenschaftskommunikation: Chancen und Risiken für die medizinische Versorgung

Digitale Medien prägen zunehmend die gesellschaftliche Realität: Nahezu jeder hat heute Zugang zum Internet, die durchschnittliche tägliche Bildschirmzeit in Deutschland beträgt über fünf Stunden, und 77,6 % der Bevölkerung nutzen aktiv Social Media (SM).^74^, ^75^ Statistisch verbringen Menschen inzwischen mehr Zeit vor Bildschirmen als in persönlichen Gesprächen mit Freunden oder Familie. Diese Entwicklung verändert nicht nur unser soziales Miteinander, sondern auch die Rolle des Arztes und die Arzt‐Patient‐Beziehung tiefgreifend.^76^, ^77^

Medizinisches Wissen ist heute in einer nie dagewesenen Breite verfügbar. Informationen sind frei zugänglich – über wissenschaftliche Blogbeiträge, Patientenportale, Online‐Enzyklopädien, Podcasts und Plattformen wie PubMed – und werden zunehmend über SM vermittelt. TikTok, Instagram, YouTube sowie Streaming‐Dienste wie Spotify oder Apple Podcasts spielen dabei eine zentrale Rolle. Patienten bewegen sich in einer Informationslandschaft, die von einer „Überinformation“ geprägt ist.

Gesundheitsrelevante Entscheidungen werden nicht mehr ausschließlich in der Arztpraxis getroffen, sondern zunehmend im Diskurs auf SM‐Plattformen. Fragen zu Lebensstil („Veganismus“) oder komplexen medizinischen Entscheidungen („Kortisonangst“, Biologika‐Therapie) werden öffentlich und kontrovers diskutiert – oft von Laien, jedoch mit erheblichem Einfluss auf das individuelle Gesundheitsverhalten.

Dies führt zu einer grundlegenden Verschiebung der ärztlichen Rolle:

Immer mehr Patienten informieren sich bereits vor dem Arztbesuch über Symptome, Diagnostik und Therapien. So tauschten sich 2021 bereits 80 % aller Krebspatientinnen und Patienten über SM aus.^78^

Ärztinnen und Ärzte sind heute seltener reine Wissensvermittler, sondern vielmehr Experte für die Validierung, Einordnung und Kontextualisierung von Wissen.

Fragen, Erwartungshaltung und Therapiezufriedenheit hängen zunehmend davon ab, welche Inhalte zuvor über soziale Medien konsumiert wurden.^78^ Problematisch ist dabei insbesondere die Verbreitung von Falschinformationen sowie der Missbrauch ärztlicher Identitäten. Gefälschte Videos und Deepfakes nutzen Namen und Bild von Ärztinnen und Ärzten ohne deren Wissen, um Aussagen zu verbreiten oder Produkte wie Nahrungsergänzungsmittel zu bewerben, häufig unter Verstoß gegen das Heilmittelwerbegesetz (HWG).

Als Reaktion entstehen erste Ansätze zur digitalen Verifikation. Blockchain‐basierte Authentifizierungssysteme sollen Nutzern künftig helfen, echte Inhalte von Fälschungen zu unterscheiden. Diese Verfahren sind jedoch energieintensiv und derzeit nur begrenzt skalierbar.^79^

Für die ärztliche Praxis bedeutet das, dass digitale Gesundheitskompetenz zu einer Schlüsselqualifikation geworden ist. Social Media kann ein wertvolles Werkzeug sein, um evidenzbasierte Informationen patientennah zu vermitteln. Gleichzeitig erfordert diese Entwicklung eine aktive Auseinandersetzung mit Chancen und Risiken, insbesondere im Hinblick auf Fehlinformationen, Erwartungsmanagement und die Integration wissenschaftlicher Evidenz in die öffentliche Diskussion.

Die Verbreitung von Falschinformationen und der Missbrauch ärztlicher Identitäten durch Deepfakes auf Social Media stellen wachsende Risiken für die Patientensicherheit und das Vertrauensverhältnis dar.

## VERSORGUNG UND PRAXISORGANISATION

Die beschriebenen digitalen Werkzeuge verdeutlichen, wie Patientinnen und Patienten zunehmend aktiv in ihre Versorgung eingebunden werden. Damit diese Innovationen jedoch ihr volles Potenzial entfalten können, bedarf es begleitender struktureller Anpassungen in Praxis und Klinik. Im Folgenden wird daher dargestellt, wie digitale Lösungen, von Praxisorganisation bis Teledermatologie, in bestehende Versorgungsstrukturen integriert werden können.

### Digitale Praxisführung in der Dermatologie

Die Dermatologie gilt traditionell als innovationsaffin, wobei digitale Verfahren wie die computergestützte Videodermatoskopie lange vor der COVID‐19‐Pandemie zur klinischen Routine gehörten.^81^ Durch neue Digitalgesetze wie das Patientendaten‐Schutz‐Gesetz (PDSG) oder das Digital‐Gesetz (DigiG) hat sich dieser Trend weiter verstärkt. Dennoch ist die flächendeckende Implementierung der Teledermatologie in Deutschland bislang unvollständig. Zwar wirkte die Pandemie theoretisch als Katalysator, jedoch zeigte eine bundesweite Untersuchung, dass über die Hälfte der deutschen Hautkliniken teledermatologische Anwendungen während dieser Zeit kaum oder gar nicht nutzte.^82^ Diese Diskrepanz verdeutlicht, dass technologische Verfügbarkeit allein nicht ausreicht, um die Hürden der klinischen Regelversorgung zu überwinden.

### Digitale Terminvergabe als Organisationsgrundlage

Digitale Terminbuchungssysteme haben sich als ein zentrales Instrument zur Entlastung von Praxisteams etabliert. Neben der reinen Buchungsfunktion erlauben sie: automatisierte Terminerinnerungen per SMS oder E‐Mail, Wartelistenmanagement zur kurzfristigen Vergabe frei werdender Termine, digitale Nachbesetzung bei Absagen, sowie die Möglichkeit, Patientinnen und Patienten mit wiederholten unentschuldigten Terminausfällen zu sperren, um zukünftige Terminausfälle zur reduzieren. So ist eine Auslastung von 90 % und mehr in den Praxen möglich, unentschuldigte Terminausfälle können signifikant reduziert,^80^, ^83^ und die Zufriedenheit auf Seiten von Patientinnen und Patienten und Mitarbeitenden erhöht werden.^84^ Darüber hinaus lassen sich im Rahmen des Buchungsprozesses gezielt Informationen zu notwendigen Unterlagen, vorbereitenden Untersuchungen und Wartezeiten bereitstellen.

Digitale Anamnesetools steigern die Effizienz, indem sie Dopplungen vermeiden und Termine strukturiert vorbereiten.

### Digitale Anamnese und Aufklärung

Ein weiterer Baustein der Digitalisierung ist die strukturierte digitale Anamnese. Patientinnen und Patienten füllen standardisierte Fragebögen bereits vor dem Termin über einen Weblink oder auf einem Tablet in der Praxis aus. Die erhobenen Informationen stehen über entsprechende Schnittstellen zeitnah im Praxisverwaltungssystem zur Verfügung und können flexibel an individuelle Parameter wie Versicherungsstatus, Buchungsgrund oder Alter angepasst werden.

### Organisatorische Entlastung durch asynchrone Kommunikation (Patient‐zu‐Arzt)

Ergänzend zur Terminbuchung lassen sich Routineanfragen durch asynchrone teledermatologische Tools („Store‐and‐Forward“) effektiv vom regulären Praxisbetrieb entkoppeln. Studien belegen, dass 80–90 % dieser Anfragen rein teledermatologisch abgeschlossen werden können, was zu einem signifikanten Zeitgewinn im Behandlungsablauf führt.^85^, ^86^ Die Vorteile bestehen in einer effizienten Triage sowie einer vollständig digitalen Abwicklung von Bagatellfällen und Verlaufskontrollen.^87^ Ein limitierender Faktor bleibt jedoch die aktuelle Vergütungssituation: Für die asynchrone Teledermatologie existiert im Einheitlichen Bewertungsmaßstab (EBM) der Gesetzlichen Krankenversicherung (GKV) bislang keine flächendeckende Abrechnungsziffer, sodass diese Leistung für GKV‐Patienten in der Regelversorgung oft nicht erstattungsfähig ist.

### Digitale Zuweisungssteuerung durch Arzt‐zu‐Arzt‐Konsile

Ein oft unterschätzter Hebel zur Entlastung dermatologischer Praxen ist die Implementierung digitaler Konsile zwischen Haus‐ und Fachärztinnen und ‐ärzten („Doctor‐to‐Doctor“). Abgebildet über den Einheitlichen Bewertungsmaßstab (EBM) oder regionale Strukturverträge (beispielsweise Lösungen wie Omnidoc ^88^ oder das sächsische Modellprojekt „eDerm“^89^) fungieren diese Systeme als effizienter Triage‐Filter vor der eigentlichen Terminvergabe. Durch die asynchrone Beurteilung hausärztlicher Anfragen können unnötige Überweisungen vermieden und dringliche Fälle priorisiert werden. Ein zusätzlicher Effekt dieser verbesserten Schnittstellenkommunikation ist ein kontinuierlicher Wissenstransfer, der die dermatologische Kompetenz der zuweisenden Hausärztinnen und Hausärzte nachhaltig stärkt.

### Online‐Rezeption als Einstiegslösung

Als Online‐Rezeptionen werden Portale bezeichnet, die verschiedene digitale Dienste wie Terminbuchung, Rezeptanforderungen oder den Abruf von Befunden bündeln. Solche Lösungen eignen sich insbesondere für Praxen mit bislang geringem Digitalisierungsgrad, bergen jedoch die Gefahr redundanter Prozesse und paralleler Strukturen. Daher empfiehlt sich eine strategische Auswahl digitaler Werkzeuge entlang eines klar definierten Patientenpfades.

### Elektronische Patientenakte (ePA)

Mit der verpflichtenden Einführung der elektronischen Patientenakte (ePA) für alle Versicherten eröffnen sich neue Chancen für eine sektorenübergreifende Versorgung. Sofern kein Widerspruch erfolgt, können medizinische Befunde einrichtungsübergreifend eingesehen werden.^90^ Der Zugriff für Ärztinnen und Ärzte ist dabei an einen bestehenden Behandlungskontext gebunden, der in der Regel durch das Einlesen der elektronischen Gesundheitskarte (eGK) legitimiert wird.^91^ Um eine echte Interoperabilität zu gewährleisten, müssen Befunde in standardisierten Formaten wie den Medizinischen Informationsobjekten (MIOs) vorliegen, damit sie systemübergreifend verarbeitet werden können.^92^Technische Voraussetzung auf Praxisseite ist ein aktuelles Praxisverwaltungssystem mit ePA‐Modul sowie der Anschluss an die Telematikinfrastruktur.

### Digitale Transformation gemeinsam gestalten

Der Erfolg der digitalen Transformation hängt maßgeblich von der Einbindung des gesamten Praxisteams ab. Schulungen, transparente Kommunikation und die aktive Einbindung von Medizinischen Fachangestellten (MFA) in digitale Prozesse steigern die Akzeptanz und reduzieren Ängste.

Digitalisierung ist kein Selbstzweck – sie entlastet, wenn Mitarbeitende den Nutzen für sich selbst erkennen.

Programme wie die „Digimanager‐Ausbildung“ der Kassenärztlichen Vereinigung Westfalen‐Lippe (KVWL) unterstützen Praxen und Praxisinhaber aktiv im Transformationsprozess.

Über die Praxisorganisation hinaus eröffnet die Teledermatologie die Möglichkeit einer ortsunabhängigen, flexiblen Versorgung.

Technologische Verfügbarkeit allein reicht nicht aus, um die Hürden der klinischen Regelversorgung zu überwinden; es bedarf begleitender struktureller Anpassungen in Praxis und Klinik.

### Teledermatologie

Teledermatologie hat sich in den letzten Jahren zu einem essenziellen Bestandteil der dermatologischen Versorgung entwickelt – angetrieben durch technologische Fortschritte und den wachsenden Bedarf an dermatologischen Leistungen.^93^ Einen entscheidenden Wendepunkt in der teledermatologischen Versorgung markierte die COVID‐19‐Pandemie. Durch die umfassenden Kontaktbeschränkungen, die sowohl das private als auch das medizinische Umfeld betrafen, kam es zu einem deutlichen Rückgang der dermatologischen Versorgung. Kompensatorisch kam es zu einer Ausweitung telemedizinischer Angebote, um die dermatologische Patientenbetreuung ‐ zumindest teilweise – aufrechtzuerhalten.^94^ Die Bedeutung der Teledermatologie wird durch die bestehende deutschsprachige S2k‐Leitlinie unterstrichen,^95^ die den festen Stellenwert dieses Versorgungsbereichs verdeutlicht und sich aktuell in Überarbeitung befindet.

In der Teledermatologie lassen sich vorrangig synchrone und asynchrone Verfahren unterscheiden; ergänzend finden sich kombinierte Verfahren und Hybridmodelle.

Bei der synchronen Teledermatologie erfolgt der direkte Austausch zwischen Patientinnen und Patienten und Ärztinnen und Ärzte in Echtzeit, meist über eine gesicherte Videoverbindung. Die Live‐Videosprechstunde ermöglicht eine unmittelbare Anamnese, visuelle Begutachtung und Therapieempfehlung. Besonders in der ambulanten Versorgung eröffnet dies Vorteile wie die Reduktion von Anfahrtswegen, erweitere Möglichkeiten für Patientinnen und Patienten mit eingeschränkter Mobilität, flexiblere Termine und eine niedrigere Infektionsgefahr. Gleichzeitig bleiben Rückfragen unmittelbar möglich. Limitationen bestehen bei der Beurteilung diskreter Hautveränderungen, insbesondere solcher, die eine hochauflösende Fotodokumentation oder dermatoskopische Untersuchung erfordern. Die asynchrone Teledermatologie hingegen basiert auf dem Store‐and‐Forward‐Prinzip. Hierbei werden klinische Angaben und Bildmaterial übermittelt und zeitversetzt durch dermatologische Ärztinnen und Ärzte begutachtet. Ein Beispiel ist die am Universitätsklinikum Leipzig AöR implementierte Lösung für klinikinterne Telekonsile. Dabei erfassen die konsilstellenden Ärztinnen und Ärzte Fotos über eine spezialisierte App, die an das Krankenhausinformationssystem angebunden ist. Die dermatologische Beurteilung erfolgt anschließend ohne direkten Patientenkontakt, was eine flexible Einbindung in den Klinikalltag ermöglicht. In einer Evaluation dieser Methode über den Zeitraum Februar bis Juli 2023 wurden 21,5 % (90 von 419) aller dermatologischen Konsile teledermatologisch bearbeitet.^96^ Seitens der konsiliarisch tätigen dermatologischen Ärztinnen und Ärzte konnte in 92,7 % dieser Fälle die Fragestellung vollständig telemedizinisch gelöst werden; nahezu die Hälfte berichtete zudem von einer Zeitersparnis von 30‐60 Minuten pro Konsil. Neben der Entlastung ärztlicher und pflegerischer Ressourcen konnten Patiententransporte und damit Infektionsrisiken reduziert werden.

Eine Evaluation klinischer Telekonsile zeigte, dass über 90 % der Anfragen vollständig telemedizinisch gelöst werden konnten, bei einer Zeitersparnis von bis zu 60 Minuten pro Konsil.

Ein weiterer innovativer Ansatz ist der Einsatz KI‐gestützter Chatbots in der Teledermatologie. Diese Systeme können erste Symptomabfragen durchführen, strukturierte Anamnesedaten erheben und basierend auf trainierten Algorithmen Verdachtsdiagnosen formulieren.

Ein etabliertes Beispiel für diese Technologie ist Ada Health, das bereits 2011 von Dr. Claire Novorol, Professor Martin Hirsch und Daniel Nathrath gegründet wurde. Die Anwendung, die bis heute in den App‐Stores verfügbar ist und allein im Google Play Store über zehn Millionen Downloads verzeichnet, verdeutlicht die hohe Akzeptanz solcher Systeme.^97^

So können ärztliche Entscheidungsprozesse gezielt vorbereitet und die Fallbearbeitung effizient unterstützt werden. Insbesondere im Zusammenspiel mit asynchronen Telekonsilen können Chatbots dazu beitragen, standardisierte Bild‐ und Befunddaten zu erfassen und so die Qualität der dermatologischen Beurteilung zu verbessern. Eine aktuelle Studie von Shapiro et al. zeigte, dass ein auf ChatGPT‐4 basierender Chatbot in über 70 % der Fälle die korrekte Diagnose stellte.^98^ Zudem lieferte dieser in 84 % der Fälle präzise Bildbeschreibungen, deren Qualität jene der Ärztinnen und Ärzte übertraf. Dies verdeutlicht das erhebliche Potenzial KI‐gestützter Systeme für die zukünftige dermatologische Versorgung.

Teledermatologie kann sowohl im ambulanten als auch im stationären Setting einen erheblichen Beitrag zur Versorgungsqualität leisten.

**ABBILDUNG 4:**
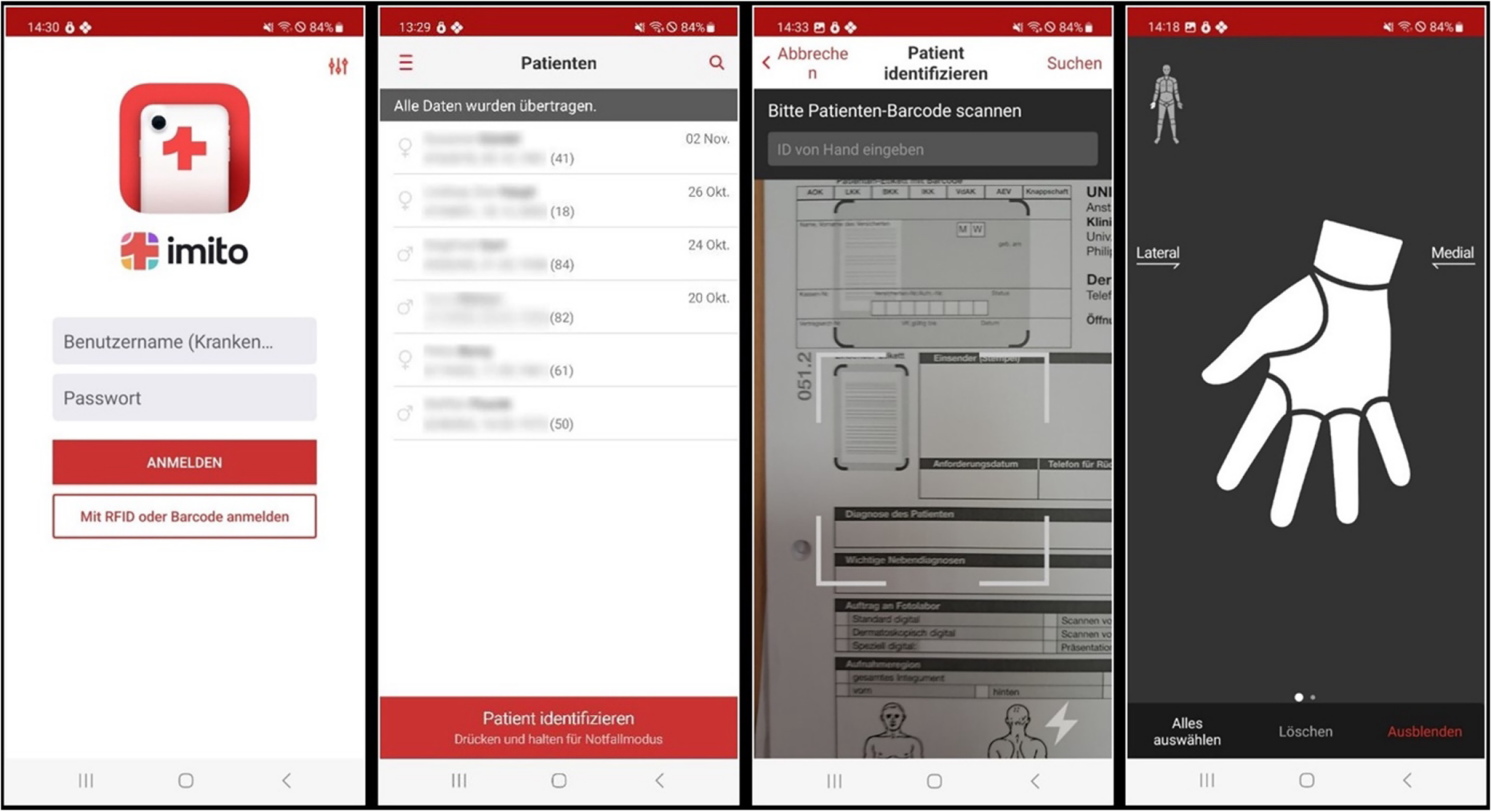
Benutzeroberfläche der imito‐App zur klinischen Fotodokumentation. Die Abfolge zeigt (v. L. n. r.): den Login‐Bereich, die Patientenliste des angeschlossenen Krankenhausinformationssystems (KIS), den Scan‐Vorgang eines Barcodes zur Patientenzuordnung sowie die anato‐mische Lokalisation (Body‐Mapping) zur präzisen Markierung der betroffenen Hautstelle. Die App ermöglicht so einen strukturierten, asynchronen Teledermatologie‐Workflow.

Zukünftig bietet die Kombination aus klassischen teledermatologischen Verfahren und unterstützenden KI‐Systemen weitere Möglichkeiten, um die dermatologische Versorgung noch effizienter und patientenzentrierter zu gestalten (Abbildung 4).

### Virtuelle Patientenpfade in der Dermatologie

Die dermatologische Versorgung in Deutschland ist derzeit überwiegend analog organisiert und erfordert von Patientinnen und Patienten eine eigenständige Navigation durch das Gesundheitssystem. Dies umfasst die Wahl des ersten Anlaufpunktes (Hausarzt, Facharzt, Klinik, Notaufnahme) sowie die Einschätzung der Behandlungsdringlichkeit. Angesichts einer begrenzten Zahl an Dermatologinnen und Dermatologen kommt es häufig zu einer Überlastung der Facharztkapazitäten durch unkomplizierte Hautbefunde wie Verrucae, Tinea oder Nävi, während komplexe Fälle nicht adäquat priorisiert werden können.

Virtuelle Patientenpfade verbinden analoge und digitale Versorgungselemente und bieten strukturelle Ansätze für ein verbessertes Patientenmanagement. Digitale Instrumente wie standardisierte Anamneseerhebung, symptom‐ und scorebasierte Befunderfassung, Integration von Wearables, Bilddokumentation, Patientenaufklärung, Anleitung zum Selbstmanagement sowie automatisierte Triage werden bislang in der Dermatologie nur eingeschränkt genutzt.^99^, ^100^

Die Deutsche Dermatologische Gesellschaft (DDG) und die Digitale Dermatologie e. V. entwickeln derzeit standardisierte und zertifizierte virtuelle Patientenpfade als Ergänzung zu Präsenzkontakten. Diese sollen primär zur Erstbeurteilung (Priorisierung, Zuweisung) sowie zur Verlaufskontrolle bei chronischen Dermatosen, Wunden, postoperativen Verläufen, onkologischen Therapien, Juckreiz und Schmerz eingesetzt werden. Geplante Indikationspfade umfassen unter anderem Psoriasis, atopisches Ekzem/Handekzeme, Prurigo nodularis/Pruritus, Akne inversa, Urtikaria, nicht‐chirurgisch behandelte aktinische Keratosen/Basalzellkarzinome, Zoster, dermatochirurgische Nachsorge und dermatoonkologische Systemtherapien.

Das Konzept sieht vor, dass Patientinnen und Patienten über einen Barcode Zugang zu einer App erhalten, über die sie krankheitsbezogene Fragen beantworten, Scores erheben, Bilder hochladen und krankheitsspezifische Informationen abrufen können. Ärztliche Behandler erhalten automatisierte Berichte und Warnmeldungen bei relevanten Veränderungen, um eine kontinuierliche statt nur intervallbasierter Betreuung zu ermöglichen.

Ergänzend bieten Erinnerungsfunktionen zur Medikation und Informationen zur begleitenden Hautpflege eine Stärkung der Adhärenz und Prävention. Die zeit‐ und ortsunabhängige Nutzung sowie die sektorübergreifende Vernetzung aller beteiligten Gesundheitsdienstleister ermöglichen eine optimierte Ressourcennutzung. Darüber hinaus liefern im Rahmen dieser Pfade erhobene Real‐World‐Daten wertvolle Grundlagen für die Versorgungsforschung.

Virtuelle Patientenpfade ermöglichen eine automatisierte Klassifikation, Priorisierung, Steuerung und das Monitoring von Hauterkrankungen.

Zusammenfassend bieten virtuelle Patientenpfade das Potenzial, die dermatologische Versorgung evidenzbasiert, effizient und patientenzentriert weiterzuentwickeln.

## FAZIT UND AUSBLICK

Die Digitalisierung hat die Dermatologie bereits nachhaltig verändert und wird die fachliche Entwicklung auch in den kommenden Jahren prägen. Die in diesem CME‐Artikel beschriebenen Beispiele zeigen die Vielfalt digitaler Innovationen, von Datenanalysen und computergestützten Diagnosesystemen über patientennahe Werkzeuge wie tragbare Messgeräte und digitale Avatare bis hin zu neuen Formen der Praxisorganisation, telemedizinischen Konsilen und virtuellen Patientenpfaden.

Diesen Ansätzen ist gemeinsam, dass sie das Potenzial besitzen, die Versorgungsqualität zu verbessern, die Beteiligung von Patientinnen und Patienten zu stärken und die Arbeit der Ärztinnen und Ärzte gezielt zu unterstützen. Gleichzeitig sind kritische Rahmenbedingungen, valide Daten, digitale Kompetenzen und eine verantwortungsvolle Einbindung in den klinischen Alltag unerlässlich.

Fachliche Netzwerke und Plattformen, wie zum Beispiel die Arbeitsgemeinschaft Digitale Dermatologie innerhalb der DDG, können dabei helfen, digitale Projekte sachlich zu begleiten, Erfahrungen aus Praxis und Forschung zu verknüpfen und eine praxisnahe Umsetzung zu fördern.

Insgesamt zeigt sich, dass die Dermatologie durch die reflektierte und interdisziplinär begleitete Integration digitaler Lösungen ihr diagnostisches, therapeutisches und organisatorisches Potenzial erweitern kann. Innovationen werden dann erfolgreich, wenn medizinische Expertise, technisches Wissen und strukturierte Zusammenarbeit Hand in Hand gehen.

## DANKSAGUNG

None.

Open access funding enabled and organized by Projekt DEAL.

## INTERESSENKONFLIKT

H.A. Haenssle erhielt Honorare und/oder Reisekostenerstattungen von Unternehmen, die an der Entwicklung von Geräten zur Hautkrebsvorsorge beteiligt sind: Scibase AB, FotoFinder Systems GmbH, Heine Optotechnik GmbH, Magnosco GmbH.

M. Tischler ist medizinischer Leiter bei der OnlineDoctor 24 GmbH und Sprecher für Doctolib.

L. Henkel, A. Bamarni, S.A. Braun, V. Busik, S. Rietz, P. Schmidle, S. Schuh, A. Seitz, S. Sitaru, S. Traidl, F. von Krogh, J. Welzel, J.K. Winkler, A.S. Vollmer, K.S. Kommoss und A. Zink haben keine Interessenkonflikte.

## [CME Questions / Lernerfolgskontrolle]

1. Welche Aussage trifft auf KI‐Klassifikationsalgorithmen zu?

- Ein Algorithmus kann grundsätzlich mit allen Datentypen (Bilder, Text, Audio etc.) umgehen, da alle Daten im Computer intern als Zahlen vorliegen.
- Bei KI‐Klassifizierungsalgorithmen ist in nächster Zeit mit großen Performancesprüngen aufgrund neuer Modellarchitekturen zu rechnen.
- Vor Anwendung in der Praxis muss man sich vergewissern, dass man den richtigen Datentyp für den Algorithmus wählt (z. B. klinisches oder dermatoskopisches Bild).
- KI‐Algorithmen eignen sich NICHT, um strukturierte Daten z. B. das abgebildete Körperteil in klinischen Bildern aus großen Datenbanken zu erfassen.
- Neue, durch KI generierte (=synthetische) Bilddaten eignen sich NICHT zum Trainieren neuer Algorithmen.

2. Worin liegt der praktische Nutzen von Heatmaps bei der KI‐gestützten BCC‐Diagnostik?

- Sie erleichtern die Bestimmung der Eindringtiefe des Tumors.
- Sie zeigen die Hautpigmentierung in unterschiedlichen Farbspektren.
- Sie markieren Areale mit hoher Tumorwahrscheinlichkeit farblich.
- Sie ermöglichen die Darstellung von Gefäßen in Echtzeit.
- Sie reduzieren die benötigte Laserleistung des LC‐OCT‐Geräts.

3. Welche Aussage beschreibt am zutreffendsten den PRO‐Score in der LC‐OCT‐gestützten AK‐Diagnostik?

- Er klassifiziert den Grad der dermalen Vaskularisierung.
- Er bewertet die basale epidermale Proliferation in definierten Stufen.
- Er misst die Dicke des Stratum corneum.
- Er ordnet Läsionen nach Risikogruppen für BCC ein.
- Er zeigt die Intensität des Entzündungsprozesses im Dermisbereich an.

4. Welche Aussagen treffen für die digitale Dermatopathologie nicht zu?

- Erste KI‐Algorithmen sind bereits CE‐zertifiziert und können somit in der pathologischen Routinediagnostik eingesetzt werden.
- Die Ausbildung der dermatopathologischen Nachwuchses wird zunehmend auch in den digitalen Raum verlagert.
- Bestimmte Forschungszweige in der Dermatopathologie beschäftigen sich aktuell mit der Integration von visueller und Textinformation in sogenannten Language‐Vision‐Modellen.
- Hohe Hard‐ und Softwarekosten beeinflussen die Geschwindigkeit der Digitalisierung in der Dermatopathologie in keiner Weise.
- Durch zunehmende Digitalisierung in dermatopathologischen Laboren und Einführung eines digitalen Workflows kann auch auf organisatorischer Ebene die Effizienz deutlich gesteigert werden.

5. Welche rechtliche Grundlage ist trotz Avatar‐Aufklärung verbindlich?

- DSGVO Art. 17 – Recht auf Löschung.
- §630e BGB – Pflicht zum mündlichen Aufklärungsgespräch.
- GOÄ §12 – Vergütungsregelung.
- TMG §5 – Impressumspflicht.
- Keine, da Avatare nicht geregelt sind.

6. Welche Aussage zu einer visuellen diagnostischen künstlichen Intelligenz (KI) trifft zu?

- Eine visuelle KI lernt effektiv durch soziale Interaktion und Diskussion (social learning).
- Der Diagnose einer visuellen KI kann immer vertraut werden, weil sie keine Fehler macht.
- Eine visuelle KI zeigt oft eine geringere diagnostische Leistung für seltene Diagnosen.
- Dermatoskopische Aufnahmen sind für eine visuelle KI grundsätzlich ungeeignet.
- Viele Smartphone‐APPs (Laiensysteme) können heute schon einen Arztbesuch ersetzten.

7. Welche Aussage zur Teledermatologie trifft am ehesten zu?

- Asynchrone Teledermatologie setzt ausschließlich auf Live‐Videogespräche und erlaubt keine zeitversetzte Beurteilung.
- Es gibt bisher keine deutschsprachige Teledermatologie‐Leitlinie.
- Teledermatologie ist aufgrund technischer Limitationen grundsätzlich nicht in der stationären Versorgung einsetzbar.
- Synchrone Teledermatologie ermöglicht eine direkte Arzt‐Patienten‐Interaktion in Echtzeit, z. B. per Videoverbindung.
- Teledermatologie hat keine Limitationen und ersetzt die klassische dermatologische Versorgung vollständig.

8. Welches primäre Ziel verfolgen die von der Deutschen Dermatologischen Gesellschaft (DDG) und Digitale Dermatologie e. V. entwickelten standardisierten virtuellen Patientenpfade?

- Ersetzung sämtlicher Präsenzkontakte in der dermatologischen Versorgung.
- Primärversorgung von Patientinnen und Patienten mit komplizierten Hauterkrankungen in der Notaufnahme.
- Erstbeurteilung (Priorisierung, Zuweisung) und Verlaufskontrolle bestimmter dermatologischer Indikationen.
- Entwicklung neuer medikamentöser Therapien für chronische Hauterkrankungen.
- Etablierung einer flächendeckenden Hautkrebs‐Screeningpflicht.

9. Welches der folgenden digitalen Instrumente wird im Rahmen virtueller Patientenpfade nicht explizit genannt?

- Standardisierte Anamneseerhebung.
- Symptom‐ und scorebasierte Befunderfassung.
- Integration von Wearables.
- Virtuelle Realität zur Therapie chronischer Schmerzen.
- Automatisierte Triage.

10. Welche zusätzliche Funktion der App im Rahmen der virtuellen Patientenpfade soll besonders die Therapietreue (Adhärenz) unterstützen?

- Automatische Hautkrebsdiagnose mittels KI.
- Erinnerung an Medikation und Anleitung zur Hautpflege.
- Verpflichtende wöchentliche Videosprechstunde.
- Bereitstellung von Laborwerten in Echtzeit.
- Steuerung der Terminvergabe in dermatologischen Kliniken.

Liebe Leserinnen und Leser, der Einsendeschluss an die DDA für diese Ausgabe ist der 30. September 2026.

Die richtige Lösung zum Thema Die Kaltplasma‐Technologie in der Behandlung von Menschen mit chronischen Wunden in Heft 2/2026 ist: 1c, 2b, 3e, 4a, 5b, 6b, 7a, 8b, 9c, 10b

Bitte verwenden Sie für Ihre Einsendung das aktuelle Formblatt auf der folgenden Seite oder aber geben Sie Ihre Lösung online unter http://jddg.akademie-dda. de ein.
